# Supplementary material for: MicroRNAs Associated with the Pathophysiological Mechanisms of Gestational Diabetes Mellitus: A Systematic Review for Building a Panel of miRNAs
Source: J Pers Med. 2023 Jul 11;13(7):1126. doi: 10.3390/jpm13071126 (PMC10381583; doi:10.3390/jpm13071126)
Supplement: Supplementary file 1 [file jpm-13-01126-s001.zip › jpm-2479034-supplementary.pdf]

PROSPERO
International prospective register of systematic reviews

Evaluate which MicroRNAs are associated with the pathological mechanisms of Gestational Diabetes Mellitus (GDM), analyzing patients worldwide.

Pedro Silva, Angela Silva, Rodrigo Santos

To enable PROSPERO to focus on COVID-19 submissions, this registration record has undergone basic automated checks for eligibility and is published exactly as submitted. PROSPERO has never provided peer review, and usual checking by the PROSPERO team does not endorse content. Therefore, automatically published records should be treated as any other PROSPERO registration. Further detail is provided **here**.

Review methods were amended after registration. Please see the revision notes and previous versions for detail.

Citation

Pedro Silva, Angela Silva, Rodrigo Santos. Evaluate which MicroRNAs are associated with the pathological mechanisms of Gestational Diabetes Mellitus (GDM), analyzing patients worldwide.. PROSPERO 2021 CRD42021291791 Available from: https://www.crd.york.ac.uk/prospero/display_record.php?ID=CRD42021291791

Review question

Evaluate which MicroRNAs are associated with the pathological mechanisms of Gestational Diabetes Mellitus (GDM), analyzing patients worldwide.

Searches

The following electronic bibliographic databases were accessed: Web of Science (https://www.periodicos.capes.gov.br/?option=com_pcollection&mn=70&smn=79&cid=81), PubMed (https://www.ncbi.nlm.nih.gov/PubMed/) and BVS Regional Portal (https://bvsalud.org/). A systematic literature search was performed to identify studies that evaluated which MicroRNAs are associated with GDM. The Web of Science, PubMed and BVS Regional Portal databases will be search from December 2021.

The studies were restricted to English, Portuguese and Spanish. Searches will be repeated before final statistics and new studies retrieved for inclusion.

Types of study to be included

It includes case-control, cohort, clinical case, cross-sectional, bibliographic reviews and systematic review studies to assess which microRNAs are associated with GDM.

Condition or domain being studied

Pregnancy changes the metabolism of carbohydrates, lipids and amino acids to create balance between fetal and maternal needs. During this period, there is an increase in insulin secretion and peripheral insulin resistance, a consequence of the secretion of “diabetogenic” hormones (placental lactogenic hormone, growth hormone and cortisol). When this compensatory mechanism is not enough to overcome insulin resistance, gestational diabetes mellitus (GDM) occurs.

Gestational diabetes can cause complications during pregnancy: fetal anomalies, polyhydramnios, fetal polycythemia / hypoxia, fetal death, neonatal hyperbilirubinemia, hypocalcemia and hypomagnesemia. Complications can be observed in childbirth, as premature birth, changes in the mode of delivery, shoulder dystocia or birth trauma.

MicroRNAs are small endogenous non-coding ribonucleic acids. They persist in several groups of eukaryotes and play critical roles during development and cell homeostasis. They are 19-25 nucleotides in length and regulate the translation of target messenger RNA. MicroRNAs can inhibit its translation, stabilize or induct its degradation. They regulate gene expression and are involved in controlling cell functions (differentiation, proliferation, apoptosis, metabolism). They are also involved in angiogenesis, oncogenesis and cardiovascular functions.

Therefore, the aim of this review was to assess the importance of microRNAs in development of GDM, in addition to their main target genes.

Participants/population

Woman worldwide of any ethnicity in any country in which a diagnosis of gestational diabetes mellitus has been received during a pregnancy (according to the International Association of Diabetes and Pregnancy Study Groups (IADPSG) and American Diabetes Association (ADA) recommended by the Word Health Organization (WHO), ie participants that make up the case groups.

Intervention(s), exposure(s)

Exposure of interest are patients with confirmed diagnosis of GDM.

Comparator(s)/control

The control group will not be parsed. The study relates the association of microRNAs in the pathophysiology of GDM, thus considering only the participants of the case groups.

Context

Free texts, language and publication data (Portuguese, English and Spanish, 10 years, respectively)

Main outcome(s)

We will identify the microRNAs involved in GDM in order to elucidate the pathophysiological mechanisms involved in the disease. Thus, reinforcing the role of microRNAs as promising biomarkers in molecular diagnosis and therapeutic responses.

Measures of effect

Data extraction will be performed with the identification of microRNAs, taking into account the mechanisms of which are associated, the direction of their regulation and in which tissue they were collected. Disregarding data related to animal models.

Additional outcome(s)

MicroRNAs can be obtained by different types of body fluids and thus provide more information about the pathophysiological factors associated with greater accuracy and diagnosis of GDM.

Measures of effect

Not applicable

Data extraction (selection and coding)

The title and summary of articles retrieved during the initial search will be independently reviewed by the researchers (R.S.S. and A.A.S.R) using a systematic strategy based on defined inclusion criteria. Any differences of opinion among reviewers regarding the inclusion or exclusion of an article will be discussed until consensus is reached.

Abstracts will be analyzed based on the following criteria:

(1) microRNA analysis in patients with Gestational Diabetes Mellitus.
(2) clinical trial studies, cross-sectional studies, systematic review, case-control study;
(3) publication in the last 5 years. No restrictions were imposed on the minimum sample size.
Non-data-based articles (books, theoretical articles and minor reviews) will be excluded.

All identified studies will be analyzed and duplicates removed. The PECO (Population with the problem: Woman worldwide with at least one pregnancy; Exposure: gestational diabetes mellitus; Comparator: patients which a diagnosis of gestational diabetes mellitus has been received during a pregnancy; Outcome: association of microRNAs with gestational diabetes mellitus; question of the study characterizes the search strategy. However, there are essential items that should be present in all reviews such as the clear definition of the research questions: Evaluate which MicroRNAs are associated with Gestational Diabetes Mellitus in patients worldwide.

Risk of bias (quality) assessment

The selected articles will be analyzed by two independent reviewers of the systematic review instrument (The Joanna Briggs Institute), for inclusion of each study, according to their quality. JBI's critical assessment tools assist in assessing the reliability, relevance and results of published works. Taking into consideration the type of study that will be approached, which may be analytical, case-control, cohort study, case reports, studies of systematic reviews and among others

Strategy for data synthesis

We will provide a summary of the studies included in the findings, structured around the type of pathology addressed and the type of biomarker used. We will discuss which microRNAs are associated with GDM by analyzing the relationship of pathophysiologies mechanisms.

Analysis of subgroups or subsets

We can consider an analysis based on the PECO elements and the study design characteristics used by the authors.

Contact details for further information

Pedro Silva
pedro.hcms@hotmail.com

Organisational affiliation of the review

Federal University of Goiás

Review team members and their organisational affiliations

Mr Pedro Silva. Federal University of Goiás
Angela Silva. Federal University of Goiás
Rodrigo Santos. Federal University of Goiás

Type and method of review

Epidemiologic, Systematic review

Anticipated or actual start date

01 December 2021

Anticipated completion date [1 change]

31 December 2022

Funding sources/sponsors

This work was supported by the personal resources from the research coordinators (Angela Adamski da Silva Reis, Ph.D. and Rodrigo da Silva Santos, Ph.D.)

Conflicts of interest

Language

English, Portuguese-Brazil, Spanish

Country

Brazil

Stage of review

Review Ongoing

Subject index terms status

Subject indexing assigned by CRD

Subject index terms

Diabetes, Gestational; Female; Humans; MicroRNAs; Pregnancy

Date of registration in PROSPERO

17 December 2021

Date of first submission

16 November 2021

Stage of review at time of this submission

The review has not started

Stage	Started	Completed
Preliminary searches	No	No
Piloting of the study selection process	No	No
Formal screening of search results against eligibility criteria	No	No
Data extraction	No	No
Risk of bias (quality) assessment	No	No
Data analysis	No	No

Revision note

New uptodate for the end date

The record owner confirms that the information they have supplied for this submission is accurate and complete and they understand that deliberate provision of inaccurate information or omission of data may

be construed as scientific misconduct.

The record owner confirms that they will update the status of the review when it is completed and will add publication details in due course.

Versions

17 December 2021
17 December 2021
28 July 2022

PROSPERO
This information has been provided by the named contact for this review. CRD has accepted this information in good faith and registered the review in PROSPERO. The registrant confirms that the information supplied for this submission is accurate and complete. CRD bears no responsibility or liability for the content of this registration record, any associated files or external websites.
